# Editorial: Case reports in pediatric infectious diseases 2022

**DOI:** 10.3389/fped.2023.1155075

**Published:** 2023-03-29

**Authors:** Hans Van Rostenberghe, Kazumichi Fujioka, Dimitri Van der Linden

**Affiliations:** ^1^ Department of Paediatrics, School of Medical Sciences, Universiti Sains Malaysia, Kelantan, Malaysia; ^2^ Department of Paediatrics, Hospital USM, Kelantan, Malaysia; ^3^ Department of Paediatrics, Kobe University, Kobe, Japan; ^4^ Cliniques Universitaire Saint-Luc, Woluwe-Saint-Lambert, Belgium

**Keywords:** pediatrics, infectious diseases, case report, diagnosis, rare presentations

**Editorial on the Research Topic** Case reports in pediatric infectious diseases 2022

## Introduction

Case reports of patients with unusual findings have been a source of inspiration for researchers and have contributed to improved clinical practice in the past (1). Especially in the field of infectious diseases in children, it is essential to have a broad knowledge of even the rare and unusual complications of common diseases and the common presentations of rare diseases. On top of that, the evolution in diagnostic methods has been fast and complex and reports of cases diagnosed with advanced laboratory techniques can contribute to improved diagnostic processes.

The editors of this special issue feel that in general, the scientific community, and more specifically, boards of universities deciding on promotion of staff tend to undervalue the contribution that case reports make to the advancement of new knowledge and to science as such. Pioneers in medicine were great supporters of documenting each unusual presentation of patients and sharing with a wide range of physicians, the findings and outcomes of such cases (2).

Writing a good case report is not easy though. Authors may get lost in less relevant details or present a textbook chapter in the introduction of the report, discouraging the target audience from finishing the reading of the report. A concise focused summary of literature can highlight why the particular case is so special and needs to be shared with as many doctors seeing similar patients as possible.

Another problem encountered is the lack of proper documentation of proper findings to exclude alternative diagnoses or contributing factors to the outcome of the patients. In order to write a good case report, the clinician should plan ahead and get all relevant investigations properly documented, of course with the permission of the patients or their guardian.

Editors of other journals have suggested that it may be a good idea to incorporate more training for physicians, not only during the undergraduate years but also in continuing medical education to ensure the skills of writing a good report are readily available in most doctors seeing a wide variety of cases.

Guidelines for reporting cases reports are available in literature (3), even though they are less well known and less applied than common guidelines for other types of scientific reports (e.g., consort statement for publication of randomised controlled trials).

It has been the great pleasure of the editors of this special issue to select and compile nine very relevant case reports in paediatric infectious diseases. In 4 reports, comprehensive pathogen analysis was useful for rapid diagnosis in pediatric infectious diseases, and it is desirable to examine its utilization in this area in the future. Other reports describe rare complications or unexpected diagnoses in patients with infectious diseases.

Below a concise critical review of each of the articles is presented.

## Summaries of case reports

Huang et al. present a case of a 10-year-old immunocompetent boy presenting with a 5-day history of intermittent, left-sided chest pain, having a left lung nodule caused by *Streptococcus intermedius*. The nodule was identified by chest radiography. The cultures of aspirates of the nodule were negative but the germ was identified by metagenomic next-generation sequencing. He was given proper antibiotics and did not require a surgical intervention. The authors highlight the rarity of the case and the importance of metagenomic next-generation sequencing in the diagnostic process.

Zhuang et al. reported a case of Congenital tuberculosis in a neonate following *in vitro* fertilization-embryo transfer. They performed pathogenic microorganism metagenomic analysis and isolated the *Mycobacterium tuberculosis* complex in a preterm infant poorly responding to treatment. Subsequently, maternal pelvic tuberculosis was confirmed. Since, *in vitro* fertilization (IVF) treatment and pregnancy can exacerbate latent tuberculosis, identifying maternal tuberculosis during IVF treatment using metagenomic approach can be a useful option.

Rodriguez et al. reported a case of an unusual pneumonia pathogen, detected by plasma cell free next-generation sequencing in an immunocompetent adolescent with acute respiratory distress syndrome. This case details a rapid diagnosis of legionella pneumonia causing severe acute respiratory distress syndrome (ARDS) in an otherwise healthy adolescent through plasma microbial cell-free DNA next generation sequencing (mcfDNA-NGS). Diagnosis by mcfDNA-NGS of this unexpected pathogen led to narrowing of antimicrobials and this novel technology can be a useful tool for paediatric infectious disease diagnosis.

Liao et al. reported a case of Respiratory tract infection of fatal severe human bocavirus 1 in a 13-month-old child. They reported the case of a 13-month-old boy who presented with a cough, shortness of breath, and wheezing, and who eventually died of severe pneumonia and acute respiratory distress syndrome (ARDS). They confirmed that HBoV1 was the only detected pathogen by Metagenomics next-generation sequencing (mNGS).

Zhang and Yu reported a rare case of severe and fulminant paediatric *Mycoplasma pneumoniae* in an immuno-competent child. There were severe lung lesions associated with pleural effusion, coagulopathy, diffuse alveolar haemorrhage and severe respiratory distress, requiring ventilator and intravenous extracorporeal membrane oxygenation (VV-ECMO) support. The authors want to highlight that early recognition and prompt institution of advanced life support measures the treatment can be successful.

Zhang et al. reported another rare complication of *Mycoplasma pneumoniae*: multiple cardiac thrombi and pulmonary embolism. They successfully treated the case with thrombolytic and anticoagulant therapy. While other thromboembolic complications of *Mycoplasma pneumoniae* have been reported, this may be the first report of cardiac thrombi as a complication of infection by this organism.

Han et al. reported a case of pleural empyema and necrotizing pneumonia related to methicillin resistant *Staphylococcus aureus* (MRSA) secondary infection in a teenager who initially presented with Influenza A virus infection. They describe the clinical symptoms and treatment and they highlight the importance of early recognition and application of thoracoscopy for this potentially fatal pleural empyema caused by MRSA and influenza A co-infection. The authors hope that this article will raise awareness regarding rarely occurring severe respiratory infections by MRSA in a child with normal immune function after influenza A virus infection.

Feussner et al. reported a lethal case of mastoiditis and cerebral herniation as a complication of otitis media in a 13 year old patient with Arnold Chiari Malformation. This case demonstrates a very rare lethal complication of acute otitis media on the basis of a cerebral malformation and emphasizes the need to stay alert when patients complain of symptoms after assumed resolution.

Chang et al. reported an adolescent immune-compromised patient with histoplasmosis, coming from a non-endemic area. The patient presented with nonspecific symptoms and was successfully treated with amphotericin B and itraconazole. The authors encourage a high index of suspicion for this pathogen in immune-compromised patients, even in non-endemic areas.

## Conclusion

In conclusion, the cases reported in this issue are likely to contribute to a better clinical practice and it is hoped that they are a source of inspiration for clinicians to keep sharing their special cases in excellent case reports.
